# Up and Down States and Memory Consolidation Across Somatosensory, Entorhinal, and Hippocampal Cortices

**DOI:** 10.3389/fnsys.2020.00022

**Published:** 2020-05-08

**Authors:** John J. Tukker, Prateep Beed, Dietmar Schmitz, Matthew E. Larkum, Robert N. S. Sachdev

**Affiliations:** ^1^Charité-Universitätsmedizin Berlin, Corporate Member of Freie Universität Berlin, Humboldt-Universität zu Berlin, and Berlin Institute of Health, Berlin, Germany; ^2^Neuroscience Research Center, Berlin, Germany; ^3^German Center for Neurodegenerative Diseases (DZNE), Berlin, Germany; ^4^Berlin Institute of Health, Berlin, Germany; ^5^Cluster of Excellence NeuroCure, Berlin, Germany; ^6^Einstein Center for Neurosciences Berlin, Berlin, Germany; ^7^Institut für Biologie, Humboldt Universität, Berlin, Germany

**Keywords:** Up and Down states, memory consolidation, sleep, somatosensory cortex, entorhinal cortex, hippocampus, inhibition, neuromodulation

## Abstract

In the course of a day, brain states fluctuate, from conscious awake information-acquiring states to sleep states, during which previously acquired information is further processed and stored as memories. One hypothesis is that memories are consolidated and stored during “offline” states such as sleep, a process thought to involve transfer of information from the hippocampus to other cortical areas. Up and Down states (UDS), patterns of activity that occur under anesthesia and sleep states, are likely to play a role in this process, although the nature of this role remains unclear. Here we review what is currently known about these mechanisms in three anatomically distinct but interconnected cortical areas: somatosensory cortex, entorhinal cortex, and the hippocampus. In doing so, we consider the role of this activity in the coordination of “replay” during sleep states, particularly during hippocampal sharp-wave ripples. We conclude that understanding the generation and propagation of UDS may provide key insights into the cortico-hippocampal dialogue linking archi- and neocortical areas during memory formation.

## Introduction

The nature of consciousness and its neural correlates are still not completely established. Does consciousness require the activity of a subset of neurons in the brainstem or thalamus, or is it a property reflected in the global activated states that can be observed in electroencephalogram (EEG) recordings during wakefulness? Even though the pattern of activity in EEG can be related to distinct brain states, these recordings have not elucidated the nature of consciousness. Nor have they established how or why brain states change from conscious states to sleep states. One element of awake – information acquiring – conscious brain states is that cortical neurons are depolarized, close to action potential threshold, i.e., in an activated state (Berger, 1929; Timofeev et al., 2001). While low frequency fluctuations in the EEG and membrane potential – i.e., delta rhythm (1–4Hz) – are dominant during sleep, they can also occur in awake states (Wilson and Groves, 1981; Petersen et al., 2003; Crochet and Petersen, 2006; Ferezou et al., 2007; Sachdev et al., 2015). Whether low frequency fluctuations are hallmarks of local sleep of a cortical area (Vyazovskiy et al., 2011) or reflect a state of inattention is not clear, but work in epilepsy patients suggests that low frequency epochs occur when sensory awareness is diminished (Blumenfeld, 2002; Motelow et al., 2015).

**Box 1. Models of Cortical Up and Down States**

A number of computational models have sought to elucidate mechanisms underlying Up and Down states. The older models highlighted the intrinsic single cell properties of cortical neurons and the interaction between these properties and circuit activity that could generate slow oscillations in cortex. The newer models go further and try to capture differences in the dynamics of Up and Down states during sleep and anesthesia.

In 2002, Bazhenov and colleagues suggested that a summation of PSPs, depolarization, and a persistent Na^+^ current were important in initiating transitions from Down to Up states; a Ca^2+^-dependent K^+^ current as well as synaptic depression and inhibition were important in terminating Up states (Bazhenov et al., 2002).

A model developed by Compte and colleagues suggested that the Up state was maintained by strong recurrent excitation balanced by inhibition and the transition to a Down state was due to a slow adaptation (Na^+^ dependent K^+^) current (Compte et al., 2003; see also Mattia and Sanchez-Vives, 2012).

A recent study by Jercog and colleagues modeled Up and Down states of urethane-anesthetized rats (Jercog et al., 2017). This model used adapting, balanced recurrent excitatory and inhibitory activity to reproduce a bistable membrane potential regime. The model suggests that whereas the transition from the Up to the Down state can be explained by adaptation in combination with the stochastic spiking activity during the Up state, the Down to Up transitions require external inputs.

In Levenstein and colleagues’ study, the dynamics of naturally occurring Up and Down-states in hippocampus and neocortex, as measured in the firing rates of neurons recorded from sleeping rats, could both be captured by a relatively simple model including only recurrent excitation, a slowly adapting negative feedback, and a noisy external drive (Levenstein et al., 2019). The model could best reproduce the recorded dynamics of neocortical circuits (long Up states of variable duration and shorter less variable Down states) in a regime where the Up state was stable, meaning external inputs or noise are needed to trigger a transition to a Down state. In contrast, the Down state was not stable, meaning the system spontaneously reverts back to the Up state. The recorded hippocampal dynamics were opposite (long Down states of variable duration and shorter less variable Up states, known as sharp waves), and could best be reproduced when the model was in a regime where a stable Down state needs external inputs or noise to trigger a transition to the Up state.

Finally, Nghiem and colleagues show that the dynamics of Up and Down states differ in anesthesia and slow wave sleep and that varying the adaptation strength using a cholinergic input could explain the different dynamics (Nghiem et al., 2018). Their model predicts that sensitivity of cortical dynamics to external inputs is stronger in sleep compared to anesthetics, possibly explaining why sleep is associated with memory consolidation, whereas anesthesia is generally associated with amnesia.

The Up state is a simpler, reduced model of activated, awake states; it has been called a “fragment of wakefulness” (Destexhe et al., 2007). Up states are activated states where the EEG is desynchronized, dominated by gamma frequencies, during which individual cortical neurons are depolarized close to threshold (Metherate and Ashe, 1993; Steriade et al., 1993c; Cowan and Wilson, 1994). When relating consciousness or wakefulness to Up states, there is a caveat to keep in mind: during slow wave sleep, or under anesthesia, even during these epochs of activity, the brain is not in an information acquiring state; in fact the Up states can be epochs of low responsiveness (Petersen et al., 2003; Sachdev et al., 2004; Hasenstaub et al., 2007). One other issue to bear in mind is that a majority of studies of Up and Down states (UDS) have been with anesthetics, and the slow waves that occur under anesthesia and those that occur under natural sleep have different dynamics and structure (Jercog et al., 2017; Nghiem et al., 2018; Levenstein et al., 2019; Box 1). Nevertheless, here we compare how these simple epochs of persistent activity occur in somatosensory and entorhinal cortex and how they are linked to each other and to hippocampal activity. In doing so, we consider the role of laminar structure and connectivity, intrinsic properties, neuromodulators, and inhibition in supporting UDS.

Cortical states of wakefulness, sleep, attention, and UDS have been written about extensively over the decades, with books and many reviews about the organization, mechanisms and effects of slow wave sleep (Steriade et al., 1993b; Steriade, 2001; Steriade and McCarley, 2005; Destexhe et al., 2007; Harris and Thiele, 2011; Zagha and McCormick, 2014; Poulet and Crochet, 2018). These states have been reviewed in many different contexts: (1) circuit mechanisms that generate slow rhythms and sleep and wakefulness (Steriade et al., 1993b; Steriade and McCarley, 2005; Krueger et al., 2013; Crunelli et al., 2015); (2) relationship to mechanisms of conscious processing and attention (Koch, 2004; Castro-Alamancos, 2009; Harris and Thiele, 2011; Koch et al., 2016); (3) relationship between local sleep and wakefulness, and the loss of consciousness during sleep or seizures (Blumenfeld and Taylor, 2003; Siclari and Tononi, 2017); (4) the effect of different brain states, especially sleep, slow wave sleep, and REM sleep on learning and memory and consolidation of memories (Stickgold, 2005; Stickgold and Walker, 2005; Rasch and Born, 2007; Dudai et al., 2015); (5) the role of neuromodulatory circuits of the brain including norepinephrine, acetylcholine, serotonin, dopamine, orexin, and melanocorticotrophin in changing brain states (Steriade and McCarley, 2005; Scammell et al., 2017); (6) slow rhythms in comas (Chatelle et al., 2012).

While a lot is known about the various circuits that promote sleep or wakefulness, the circuits that are causal in flipping the brain from slow wave – where the cortical EEG and the membrane potential of the individual neurons can be dominated by less than 1Hz slow waves – to REM or awake states, and vice versa, have not been elucidated. It is not known how the variety of different awake-promoting neuromodulatory circuits interact to create awake conscious states, or which mechanisms determine sleep states. Whether recurrent activity in cortical circuits is necessary to generate Up states is not known. Whether all classes of inhibitory interneurons in all cortical layers fire in phase with slow waves and Up states (Valero and Buzsáki, 2019), or whether some class of interneurons trigger the end of each Up state is still not known.

Here we begin by examining what is known about UDS in general, with a focus on the initial studies in primary sensorimotor cortical areas, and then examine what is known about these states in very differently organized interconnected cortical areas, the entorhinal and hippocampal circuits. For each of these three areas, we first describe the properties of UDS and the structure – function relationship in light of this network activity and the anatomical properties of each area. Then we review the effects of neuromodulators on UDS, particularly (but not exclusively) acetylcholine, and discuss the crucial issue of inhibition and participation of inhibitory neurons in UDS. Finally, we consider the role of UDS in cortico-hippocampal interactions and hippocampal processing during slow wave sleep, particularly focusing on sharp-wave ripples and memory consolidation.

Memory and conscious states are intimately entwined (Tulving, 1985). Conscious experience involves the formation of memories, the remembering of a past. The formation of long term memories, i.e. consolidation, is greatly benefited by sleep (for reviews see Diekelmann and Born, 2010; Chen and Wilson, 2017; Sara, 2017; Klinzing et al., 2019). During sleep hippocampal place cells have an above-chance probability to be co-active in similar sequences as during the awake state (Lee and Wilson, 2002). During slow wave sleep, neocortical activity in each Up state is associated with hippocampal sharp wave ripples (Siapas and Wilson, 1998; Sirota et al., 2003; Isomura et al., 2006; Mölle et al., 2006; Peyrache et al., 2009; Sullivan et al., 2011; Maingret et al., 2016; Headley et al., 2017). This interaction between neocortical slow rhythm of sleep, sharp wave ripples in hippocampus, and hippocampal replay is thought to be the basis of memory consolidation (for reviews, see Sirota and Buzsáki, 2005; Buzsáki, 2015; Klinzing et al., 2019; Todorova and Zugaro, 2020). For this reason, here we examine the neocortical-entorhinal-hippocampal dialogue observed through the lens of slow wave sleep and Up and Down states.

## Up and Down States in Neocortical Circuits

**FIGURE 1:**
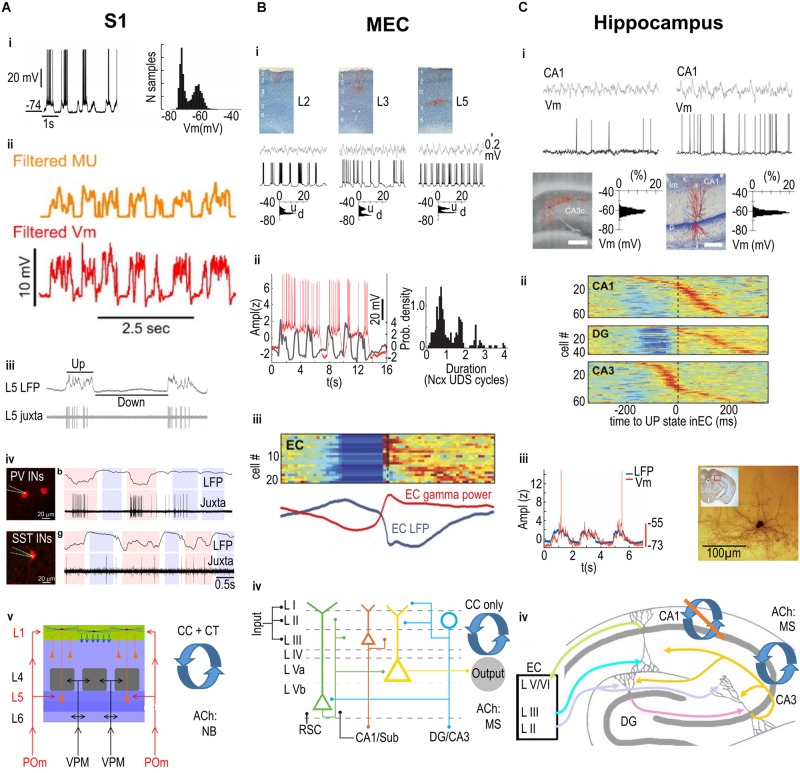
Summary of Up and Down state mechanisms across three cortical areas. **(A)** Primary somatosensory cortex (S1). i. An example intracellular recording from a neuron displaying depolarized Up states and hyperpolarized Down states (i, left panel), with a clear bimodal distribution of membrane potential values (i, right panel). ii. The multi-unit (MU) firing of neurons is correlated with the membrane potential (Vm) fluctuations. iii. Juxtacellular recording illustrating that the firing of a pyramidal neuron (in this case in layer 5; L5) is linked to the Up state recorded in the local field potential (LFP). iv. Both Parvalbumin (PV, top row) and Somatostatin (SST, bottom row) expressing interneurons (INs) preferentially fire action potentials during Up states, but also fire a minority of spikes during Down states. v. Relevant anatomical properties of S1 barrel cortex include the laminar structure (left), with layer-specific inputs from specific thalamic nuclei (POm, VPM); recurrent corticocortical (CC) and corticothalamic (CT) connectivity; and cholinergic input from the Nucleus Basalis (NB). **(B)** Medial Entorhinal Cortex (MEC). i. Intracellularly recorded principal cells in layer 2 (L2), 3 (L3), and 5 (L5), shown in top row, display depolarized Up states and hyperpolarized Down states (middle row), with a clear bimodal distribution of membrane potential values (bottom row; u = Up State, d = Down state). ii. Left panel: Intracellularly recorded Up states in a L3 pyramidal cell (red trace) can extend over several simultaneously recorded Up states in the neocortex (gray trace). Right panel: The duration of MEC L3 pyramidal cell Up states is preferentially linked to a discrete number of neocortical Up and Down state (Ncx UDS) cycles. iii. Color-coded stacked cross-correlograms of Entorhinal Cortex (EC) neurons (one per row), recorded during slow wave sleep in naturally sleeping rats. Note the cross-correlograms are aligned to the transition from Down to Up states, and also correlate with the simultaneously recorded average LFP (blue) and gamma power (red) shown below. iv. Relevant anatomical properties of MEC include the laminar structure (left), with layer-specific inputs from other cortical areas including inputs from the retrosplenial cortex (RSC) and hippocampus (CA1/Sub) to the deep layers, as well as layer-specific outputs including outputs from L2 stellate cells (blue circle) to the hippocampal areas DG/CA3 and from L3 pyramidal cells (red triangle) to CA1/Sub; recurrent corticocortical (CC) connectivity only (i.e., no corticothalamic connectivity is known); and cholinergic input from the Medial Septum complex (MS). **(C)** Hippocampus. Intracellular recordings from pyramidal cells in areas CA3 (left) and CA1 (right); the filled cells are displayed on the bottom row. Note that neither the LFP (top row) nor the membrane potential (Vm, second row) display clear Up and Down states, as shown in the unimodal distribution of Vm values (bottom row). ii. color-coded stacked cross-correlograms of DG, CA1, and CA3 neurons (one per row), recorded during slow wave sleep in a naturally sleeping rat (same rat as in panel **Biii**.), and aligned to the transition from Down to Up states. Note that there is a clear Down state only in DG (compare also to EC activity in **Biii**.). iii. Left: The intracellularly recorded membrane potential (red trace) of a CA1 interneuron fluctuates in phase with the simultaneously recorded neocortical LFP. Right: the filled interneuron from which the recording in the left panel was made, located in the “molecular layer” of CA1. iv. Relevant anatomical properties of the hippocampus, which has only a single layer of pyramidal cells extending from CA1 to CA3 (plus one layer in DG, with a strictly unidirectional connectivity), layer-specific inputs from superficial EC, and output from CA1 to deep layers of EC (other outputs not shown); recurrent connectivity is classically considered to be present amongst CA3, but not CA1 pyramidal cells; cholinergic input is from the Medial Septum complex (MS). **(Ai)** adapted by permission from Hasenstaub et al. (2007); **(Aii)** from Mease et al. (2016); **(Aiii)** from Zucca et al. (2017); **(Aiv)** from Castejon et al. (2016); **(Bi,iii,Ci,ii)** from Isomura et al. (2006); **(Bii)** from Hahn et al. (2012); **(Biv)** from Witter et al. (2017); **(Ciii)** from Hahn et al. (2006); **(Civ)** from Petrantonakis and Poirazi (2014).

While slow rhythms had been observed earlier, Steriade and colleagues were the first to recognize the relationship between the intracellular membrane potential and the cortical EEG (Steriade et al., 1993b,c,d). At about the same time intracellular recordings by Metherate and colleagues (Metherate et al., 1992; Metherate and Ashe, 1993; Cowan and Wilson, 1994) established the ubiquity of these rhythms in rodent sensory and motor cortices. This rhythm was apparent in cortical intracellular and whole cell recordings as spontaneous (less than 1 Hz) fluctuations in membrane potential, and could be observed in the EEG, local field potential (LFP), and in the multiunit activity (MUA) (Figure 1A). The terminology of Up and Down states, coined by Wilson (Wilson and Kawaguchi, 1996), arose out of the key observation that the distribution of membrane potentials for cortical neurons was bimodal: single cortical and medium spiny striatal neurons showed two stable membrane potential states, the depolarized Up state and the hyperpolarized Down state (Cowan and Wilson, 1994; Wilson and Kawaguchi, 1996). A bimodal distribution indicated that the mix of excitatory and inhibitory inputs to the neuron, and the intrinsic voltage-dependent conductances, together created two preferred membrane potentials for each neuron. From this early work, it was also clear that the Up state was a high conductance state and that in the Down state, the input resistance of neurons was higher and few neurons were actively providing synaptic input to any cortical or striatal neuron (Cowan and Wilson, 1994; Paré et al., 1998; Destexhe and Paré, 1999; Waters and Helmchen, 2006). Finally, this work and work from the McCormick lab showed that the Up state was dominated by gamma band activity and that the fast post-synaptic potentials (PSPs) in each Up state could be inhibitory, that is Up states were a balanced mixture of excitation and inhibition (Cowan and Wilson, 1994; Steriade et al., 1996; Sanchez-Vives and McCormick, 2000; Shu et al., 2003; Haider et al., 2006).

So within the slow wave sleep rhythm, two distinct brain states can be observed, one where the cortex, and ostensibly the brain are quiet, and another where cortex is in an activated, high gamma state (Destexhe et al., 2007). In the first papers describing this rhythm, the mechanisms of Up and Down states were explored in primary sensory cortical areas (Metherate et al., 1992; Metherate and Ashe, 1993; Steriade et al., 1993a,c,d; Cowan and Wilson, 1994). One idea was to examine whether these rhythms were generated intracortically, or thalamic inputs were necessary to generate the slow wave rhythms. This work indicated that even though thalamic neurons were in phase with cortical Up and Down states, large excitotoxic thalamic lesions, combined with transsectioning of the corpus callosum had no effect on the generation of the slow rhythm in parietal cortex (Steriade et al., 1993d; but see Poulet et al., 2012; Zagha et al., 2013). In fact, UDS can be elicited even in isolated brain slices (Sanchez-Vives and McCormick, 2000).

A second idea was that the brain stem arousal centers, cholinergic pedunculopontine tegmentum (PPT) and noradrenergic locus coeruleus, trigger Up states. Microstimulation in the PPT triggered persistent activity, an activated state that was evident as tonic depolarization in cortical neurons (Steriade et al., 1993a). Additionally, stimulation of the basal forebrain, another cholinergic site, elicited a desynchronization of the cortical EEG (Metherate et al., 1992; Metherate and Ashe, 1993).

Another hypothesis was that depolarizations reflected excitatory inputs, and the hyperpolarizations were a manifestation of inhibition. However, recordings from cortical pyramidal neurons *in vivo* and *in vitro* showed that the Up states were high conductance states, whereas down states were low conductance states; furthermore, the fast PSPs in the Up state membrane potential occurred in the gamma frequency, and reversed at the reversal potential of inhibition (Cowan and Wilson, 1994; Steriade et al., 1996; Shu et al., 2003; Hasenstaub et al., 2005; Haider et al., 2006; Waters and Helmchen, 2006). These experiments suggested that excitation and inhibition increased and decreased together, that the Up state was dominated by both excitation and inhibition, and the Down state was a state when cortical circuits were quiescent. In the last few years, these ideas have been investigated in greater detail in anesthesia, quiet wakefulness, and activated states.

### Up and Down States in the Somatosensory “Barrel” Cortex

The rodent barrel cortex is a model somatosensory system that has been used extensively for the study of Up and Down states. The organization, structure and function of this cortex have been extensively reviewed (Kleinfeld et al., 2006; Petersen, 2007; Diamond et al., 2008; Sachdev et al., 2012; Feldmeyer et al., 2013). Briefly, the somatosensory cortex is a prototypical primary sensory cortex, it is 6 layered and receives thalamic inputs from lemniscal and paralemniscal thalamic nuclei. The lemniscal pathways carry touch, pressure, temperature, and proprioceptive input primarily targeting layer 4 and 6, and the paralemniscal pathways target layer 1 and 5. Thalamic input is excitatory, but the excitatory input can target inhibitory interneurons (Agmon and Connors, 1991). The canonical thalamocortical circuit (reviewed in Sachdev et al., 2012) consists of excitatory input from thalamus, that targets both excitatory and inhibitory neurons in cortex, with the excitatory input driving other cortical neurons in a feedforward manner, i.e., excitation is balanced by inhibition. When excitatory inputs are active, inhibitory inputs are activated within a few milliseconds. The somatostatin cortical interneuron in layer 2/3 and 5 is an exception to this rule that inhibitory neurons in cortex are co-activated with local excitatory neurons (Gentet et al., 2012). The principal cortical neuron, the pyramidal cell is the main output neuron of cortex, and it is the source of the majority of local recurrent and long-range excitation in cortex. Pyramidal cell bodies can be found in layer 2/3, 4, 5, and 6. It is worth noting that there is no known class of intrinsically active – pacemaker – excitatory neuron in somatosensory cortex, i.e., no neurons continue to fire action potentials in the absence of synaptic input, but there may be a class of inhibitory interneuron, a somatostatin positive neuron, that can fire intrinsically (Fanselow et al., 2008; Fanselow and Connors, 2010; see also Zucca et al., 2017).

### Up and Down States in the Medial Entorhinal Cortex

The entorhinal cortex (EC) connects the parietal, temporal and prefrontal cortices to the hippocampal formation; thus, it forms the interface between brain regions coding ongoing sensory information and storing long-term memory on the one hand, and those responsible for encoding and consolidating memories. The EC serves as the primary input and output structure of the hippocampus: there are two parallel input/output channels one in the lateral and the other in the medial entorhinal cortex each connected distinctly to the hippocampus. While primary sensory neocortical areas are all intimately linked to multiple thalamic nuclei, there is no evidence of strong monosynaptic thalamic inputs to the MEC. The persistence of up down state transitions in the absence of thalamic inputs to the MEC indicates that there are several other motifs of recurrent connectivity that can provide the circuitry necessary for maintenance of UDS besides those found in the somatosensory cortex.

One consequence of the organization of the entorhinal cortex, specifically the fact that multiple neocortical areas provide excitatory long range input to this cortex, is that Up states in neocortex are likely to propagate into entorhinal cortex. Indeed, entorhinal neurons show UDS *in vivo* (Isomura et al., 2006; Hahn et al., 2012; Figures 1Bi,ii); and gamma oscillations are nested on up states (Isomura et al., 2006; Figure 1Biii). Surprisingly, even in the absence of long range input, *ex vivo* in brain slices, it is possible to generate UDS in both the lateral (Namiki et al., 2013) and medial (Mann et al., 2009; Tahvildari et al., 2012) subdivisions of the entorhinal cortices. *In vivo*, the Up states in MEC can last more than 2 s, almost double the duration of Up states in somatosensory and motor cortices (Cowan and Wilson, 1994; Sachdev et al., 2004; Hasenstaub et al., 2007; Hahn et al., 2012; Figure 1Bii; but also see Haider et al., 2006, 2013). One consequence of this long duration Up state in entorhinal cortex is that even though MEC activity is phasically linked to neocortical Up states, activity in MEC can skip one or two cycles (Hahn et al., 2012). The longer Up states in MEC potentially arise from intrinsic membrane and synaptic properties of pyramidal neurons in MEC. Pyramidal cells in MEC have persistent firing and a Kainate receptor dependent slow glutamatergic current (Digby et al., 2017). This current is not found in pyramidal cells in primary somatosensory cortex (S1).

### Laminar Pattern of Up and Down States

There are two distinct possibilities for a laminar profile of activity during slow waves. First it is possible that a class of cortical neurons in a particular layer is more responsive to inputs or is intrinsically more active, or more excitable. A second possibility is that a class of inputs to a particular layer initiates each Up state. The studies that have examined this question come to very different conclusions. In ferret brain slices, Up states begin in layer 5 (L5) and propagate from there. *In vivo* in anesthetized mice, the picture is more complex. While L5 MUA can precede activity in other layers, activity in L4 can also precede the initiation of Up states in other cortical layers (Sanchez-Vives and McCormick, 2000).

In contrast, in MEC, L3 pyramidal neurons show the strongest correlation to Up and Down states. Layer 3 neurons maintain and propagate the UDS activity in the MEC; L3 pyramidal cells are the most active population of excitatory cell types across all layers in the MEC. The strong role for L3 pyramidal neurons in maintaining and propagating the UDS activity in the MEC arises from: (1) the strong recurrent connections that target other layers (Dhillon and Jones, 2000; Winterer et al., 2017); (2) the slow decay kinetics of EPSPs that facilitate synaptic integration and spiking at lower frequencies (Gloveli et al., 1997).

Stellate cells in MEC L2 can also fire in phase with UP states, but fire much less than the L3 pyramidal neurons. This is in part due to strong inhibition of the L2 stellate cells (Beed et al., 2013), which reduces the subthreshold depolarization on individual cells during each Up state. Inhibition is also likely to bring about the Up to Down state transition which we discuss in more detail in the next section.

## Role of Inhibition and Participation of Inhibitory Neurons During UDS

### Inhibition and Up and Down States in Neocortex

One simple hypothesis regarding Down states is that they are a reflection of increased inhibition. This hypothesis implies that the balance between excitation and inhibition shifts during each Up state, and that classes of inhibitory interneurons are active at the end of the Up state and terminate the Up state. Furthermore, these neurons could continue to fire in the Down state, maintaining the hyper polarized Down state. A related hypothesis would be that a class of inhibitory neurons is intrinsically active requiring no excitatory input, or that the class of interneurons that trigger Down states are part of a distinct cortical network, that is partly out of phase with the global EEG and LFP recorded in cortex. These ideas have spurred a large number of additional investigations tracking whether interneurons all fire in phase with cortical Up states (Zucca et al., 2017, 2019; Knoblich et al., 2019; Senzai et al., 2019; Valero and Buzsáki, 2019).

As mentioned above, a large variety of cortical inhibitory interneurons, distributed throughout all cortical layers, have been described (Markram et al., 2004; Tremblay et al., 2016; Feldmeyer et al., 2018). The neurogliaform cells are the most common interneuron type found in layer 1 in S1; they are also found in layers 2–6 (Kawaguchi and Kubota, 1997; Ascoli et al., 2008; Kubota, 2014). Parvalbumin positive neurons are found in layers 2–6; they are the classical fast spiking, soma targeting (basket cells), and axon targeting (chandelier cells) neurons (Kawaguchi and Kubota, 1997; Ascoli et al., 2008). The somatostatin positive (SST) interneurons are a class of mostly non-fast spiking, dendrite targeting neurons, including the Martinotti cell found in layer 2/3 and 5/6. Non-Martinotti SST neurons can be found in layer 4 (Kawaguchi and Kubota, 1997; Wang et al., 2004; Ma et al., 2006; Xu et al., 2013; Kubota, 2014). Another important class of interneurons, characterized by the expression of vasointestinal peptide (VIP), is part of a disinhibitory circuit with the SST neurons and are found in layers 1–6 (Caputi et al., 2009; Cauli et al., 2014; Prönneke et al., 2015).

These classes of interneurons have been studied extensively especially in quietly sitting awake animals and in anesthetized animals. A large body of work shows that low frequency oscillations are often evident in the quietly sitting awake state, and that these low frequency oscillations can be abolished by movement. In rodents, whisking activates the cortex, and the cortical EEG and membrane potential of pyramidal neurons reflects this change in state (Petersen et al., 2003; Crochet and Petersen, 2006; Poulet and Petersen, 2008; Gentet et al., 2010; Pala and Petersen, 2018). During quiet wakefulness, pyramidal neurons, fast spiking interneurons, and non-fast spiking SST neurons fire in phase with the slow rhythm. Activation of the cortex by whisker motion triggers a decrease in spiking of pyramidal and parvalbumin-positive interneurons. In contrast during activated states, the somatostatin positive interneurons are persistently depolarized and increase their firing rate suggesting that in the awake state when the mice whisk, this class of interneurons is linked to a distinct class of inputs that do not drive the pyramidal neuron or the fast spiking parvalbumin neurons.

In anesthetized animals, juxtacellular recordings also show that parvalbumin and SST neurons fire mostly in phase with Up states. However, even though the activity of both PV and SST interneurons decreases during Down states, it is not completely abolished (Zucca et al., 2017, 2019). Something very similar is observed in the visual cortex of anesthetized mice (Knoblich et al., 2019). In this study, while the membrane potential of pyramidal and parvalbumin positive neurons was bimodally distributed, SST neurons could be divided into two classes, one that fired in phase with Up and Down states – its membrane potential was bimodally distributed – and the other class of SST neurons that continued firing even in Down states and its membrane potential was unimodally distributed (Knoblich et al., 2019). Another recent study shows that a population of neurons in L6 of visual cortex fired specifically in Down states (Senzai et al., 2019). The anatomical identity of these extracellularly recorded neurons is not known. Finally, in prefrontal cortex of naturally sleeping mice SST positive cells fired in phase with the slow wave sleep rhythm (Funk et al., 2017). The spontaneous firing patterns of most other classes of interneurons have not been studied in the context of Up and Down states and have not been linked to slow oscillations.

### Inhibition and Up and Down States in the Medial Entorhinal Cortex

Superficial layers (layers 2 and 3) of the MEC are embedded in a dense inhibitory network. Parvalbumin positive, perisomatic targeting inhibitory neurons strongly modulate the output of both L2 stellate and L3 pyramidal cells, thereby organizing the circuitry predominantly at oscillatory frequencies in the gamma range. This pattern of inhibition is in stark contrast to the pattern seen in somatosensory cortex, where there is as yet no evidence of differential levels of inhibition in the different cortical layers. In the MEC on the other hand, there is a strong gradient of inhibition along the dorsoventral axis. There is almost twofold stronger inhibition in the dorsal MEC compared to the inhibition at the ventral levels. This differential gradient is related to input from parvalbumin-expressing (PV) interneurons (Beed et al., 2013).

In *ex vivo* slice preparations of entorhinal cortex, it is possible to obtain Up and Down oscillatory activity in the superficial layers of the MEC (Mann et al., 2009; Tahvildari et al., 2012; Salkoff et al., 2015). Excitatory and inhibitory neurons in the layers 2 and 3 of the MEC take part during UDS (Tahvildari et al., 2012; Neske et al., 2015). Parvalbumin interneurons in layers 2 and 3 also participate in the slow rhythm and are strongly depolarized, firing action potentials in the Up state in gamma frequency range (Salkoff et al., 2015). In contrast, one subset of SST interneurons is only weakly driven by each Up state and fires few spikes (Tahvildari et al., 2012), whereas the fast spiking SST interneurons fire in phase with each Up state (Neske et al., 2015). Other work has shown that inhibition, in particular GABA-B receptor dependent inhibition, can play a role in terminating Up states (Mann et al., 2009; Craig et al., 2013). While a buildup of slow inhibitory effects is possible for terminating each Up state, no class of interneurons has shown a pattern where their activity increases over the course of an Up state, or where the neurons fire out of phase with each Up state.

There are multiple other classes of interneurons in MEC including those identified by expression of serotonin receptor type 3a (5HT3a), cholecystokinin (CCK) and VIP, but in the *ex vivo* slice model at least, these classes of interneurons are negligibly driven by each Up state (Tahvildari et al., 2012).

## Neuromodulation of UDS

### Neuromodulation of Up and Down States in the Somatosensory Cortex

As mentioned above, brainstem and hypothalamic circuits have been linked to an ascending activating system. This system is the source of many neuromodulators (reviewed in Scammell et al., 2017). Here we will focus on two classical neuromodulators, acetylcholine and norepinephrine.

#### Acetylcholine

One source of acetylcholine in the brain is the brainstem pedunculopontine nucleus that has neurons that project to thalamus, hypothalamus and basal forebrain, But the cholinergic fibers that target the somatosensory cortex arise from the nucleus basalis (McKinney et al., 1983; Mesulam et al., 1983; Eckenstein et al., 1988). Fibers immunoreactive for the acetylcholine synthetic enzyme, choline acetyltransferase or stained for the cholinergic degradative enzyme, acetylcholine esterase can be detected in all layers of the rat parietal cortex, but the innervation to laminae I–III and V is particularly dense (Kristt, 1979; Houser et al., 1985; Lysakowski et al., 1989; Umbriaco et al., 1994). In barrel cortex, the acetylcholinesterase fibers are dense in all layers except for layer 4 (Sachdev et al., 1998).

Microsimulation in the brainstem, in the pedunculopontine nuclei can trigger persistent activity, an activated state, that is evident as a tonic depolarization in cortical neurons (Steriade et al., 1993a). The effect of the stimulation in changing the cortical state, can take two pathways, one where the cholinergic PPT neurons activate thalamus (Crunelli et al., 2015), a second where the cholinergic PPT neurons activate the basal forebrain neurons. In line with the second mechanism, stimulation of the basal forebrain can also elicit a acetylcholine dependent desynchronization of the cortical EEG (Metherate et al., 1992; Metherate and Ashe, 1993).

The cholinergic PPT neurons and the basal forebrain neurons increase their activity during wakefulness (McCarley and Hobson, 1975; Lee et al., 2004; Hassani et al., 2009). One difference between the brainstem cholinergic neurons and the basal forebrain cholinergic neurons is that while 80% of the pedunculopontine cholinergic neurons fire in phase with cortical Up and Down states (Mena-Segovia et al., 2008; Ros et al., 2010; Mena-Segovia and Bolam, 2011) the basal forebrain neurons show no clear phasic activity with cortical Up and Down states (Lee et al., 2004; Hassani et al., 2009). Indeed, even though the activity of basal forebrain axons in S1 increases and is related to the generation of activated states, the effect of cholinergic activity is to oppose the movement-triggered activated and persistently depolarized state observed in barrel cortex (Eggermann et al., 2014).

#### Norepinephrine

The locus coeruleus is the exclusive source of norepinepherine in the central nervous system, with the individual axons of these neurons traversing widespread cortical areas (Dahlstroem and Fuxe, 1964; Swanson and Hartman, 1975; Sara, 2009). The noradrenergic fibers traverse all cortical layers in somatosensory barrel cortex, including layer 4 (Simpson et al., 2006).

Just like the brainstem pedunculopontine neurons, the locus coeruleus neurons fire in phase with cortical Up and Down states (Lestienne et al., 1997; Eschenko et al., 2012) and they are more active during wakefulness than during slow wave sleep (Aston-Jones and Bloom, 1981). Optogenetic stimulation of these neurons can cause an immediate transition from sleep to wakefulness (Dringenberg and Vanderwolf, 1997; Carter et al., 2010). Furthermore, in the awake and activated state, activity of the locus coeruleus neurons promotes persistent activity and a desynchronized state (Constantinople and Bruno, 2011).

One caveat with most of this work, is that even though the study of the brainstem activating system has a long history, the study of how these systems interact with each other and with the hypothalamic hypocretin/orexin and neurons is still in its infancy (Scammell et al., 2017).

### Neuromodulation of Up and Down States in the Medial Entorhinal Cortex

Acetylcholine is a key neuromodulator of the MEC. In contrast to the somatosensory cortex, which receives cholinergic inputs from the nucleus basalis, the bulk of the cholinergic input to the MEC arises from the medial septum (Lewis and Shute, 1967; Köhler et al., 1984; Gonzalez-Sulser et al., 2014; Desikan et al., 2018).

The medial septum cholinergic neurons are key players in generating theta oscillations, a marker of attention and arousal. This rhythm is absent during sleep suggesting that the cholinergic tone in the entorhinal cortex is low during slow wave sleep. Medial septum cholinergic fibers strongly modulate and innervate both excitatory and inhibitory neurons (Desikan et al., 2018). Fast and slow cholinergic currents depolarize both the SST and 5HT3a interneuron populations whereas the slow cholinergic currents mostly hyperpolarize the excitatory cells and the PV interneurons (Desikan et al., 2018). The bimodal effect of cholinergic modulation on the different cell types coupled with their respective roles during Up and Down states makes it an important neuromodulatory candidate for brain states in the MEC.

In addition, acetylcholine released from cholinergic fibers has a profound effect both on subthreshold membrane potential oscillations (Heys et al., 2010) as well as spiking activity in the MEC (Heys et al., 2012; Tsuno et al., 2013). The application of cholinergic agonists on *ex vivo* slice preparation can induce persistent firing (i.e., Up states) in pyramidal neurons in the MEC (Egorov et al., 2002; Tahvildari et al., 2012; Jochems et al., 2013). This effect of acetylcholine is unique for neurons in the entorhinal cortices and is not observed in other cortical pyramidal neurons. Although a lot is known about the *ex vivo* effects of acetylcholine, very little is known about the spatio-temporal organization of cholinergic inputs during changes in brain state in the MEC.

## Cortico – Hippocampal Interaction and Hippocampal Processing During UDS

### Memory Consolidation, Hippocampal Replay, and Sharp-Wave Ripples in UDS

Memory consolidation is a key function associated with slow wave sleep. In the hippocampus, memory consolidation has been associated with sharp wave ripples (SWRs), consisting of a high-frequency (∼150 Hz) “ripple” riding on top of a depolarizing “sharp-wave” event (see Buzsáki, 2015, for a review). SWRs appear to be generated in area CA3 and propagate via CA1 and the subiculum into the cortex (Buzsáki et al., 1983; Chrobak and Buzsáki, 1996; Csicsvari et al., 2000; Khodagholy et al., 2017; Nitzan et al., 2020). A process called “replay” has also been linked to SWRs; during replay when the animal is at rest or asleep, cell assemblies active during waking information-acquiring behaviors are reactivated (for a review, see Foster, 2017). One hypothesis is that replay strengthens synaptic connections in the neocortex, thereby mediating a “transfer” of memory from the short-term hippocampal system to the long-term neocortical system (Marr, 1971; Buzsáki, 1989; Chen and Wilson, 2017).

Evidence in support of this idea has shown that: (1) replay in hippocampus and other cortical areas is significantly correlated (Ji and Wilson, 2007; Ólafsdóttir et al., 2016; Rothschild et al., 2017); (2) interrupting hippocampal SWRs (and thus the replay events associated with them) during post-learning sleep reduces memory consolidation (Girardeau et al., 2009; Ego-Stengel and Wilson, 2010); (3) ripple activity in hippocampus is coupled to cortical activity in many brain areas (Chrobak and Buzsáki, 1994, 1996; Logothetis et al., 2012; Wang and Ikemoto, 2016; Headley et al., 2017; Khodagholy et al., 2017), including cortical replay events in the prefrontal cortex (PFC; Peyrache et al., 2009); and (4) strengthening this coupling by precisely timed SWR-locked stimulation of the PFC improves memory consolidation (Maingret et al., 2016).

During slow wave sleep, SWRs in the hippocampus appear remarkably closely linked to neocortical Up and Down states. Some studies report that the SWRs in CA1 are more prevalent during Down states (Battaglia et al., 2004), their occurrence peaking just before the Down-Up transition (see also Khodagholy et al., 2017). Other studies report that SWRs are more prevalent during Up states as defined by: (1) EEG, local field potential or multiunit activity in prefrontal cortex (PFC; Siapas and Wilson, 1998; Mölle et al., 2006; Isomura et al., 2006; Peyrache et al., 2009; Maingret et al., 2016); (2) MUA and LFP in somatosensory cortex (Sirota et al., 2003); (3) MUA in entorhinal cortex (Isomura et al., 2006; Sullivan et al., 2011) or (4) pooled MUA from several cortical sites (Headley et al., 2017). Taken together SWRs seem to be coupled to cortical Up and Down states.

This coordination between cortex and hippocampus is likely to be important for memory consolidation. A number of studies have looked into the coordination of cortical and hippocampal activity in the context of SWR propagation, memory consolidation, and replay. These studies show that both fast oscillations and unit activity in MEC and other retrohippocampal areas are coupled to hippocampal ripples (Chrobak and Buzsáki, 1994, 1996). The functional consequences of this feedback to the MEC are not known, but the inputs from CA1 and subiculum, impinging mostly on MEC L5, could support the coordinated replay between CA1 and the deep layers of MEC during sleep (Ólafsdóttir et al., 2016). In contrast, recordings from the superficial layers of MEC in awake animals reveal that replay in the MEC can also be independent of replay in CA1 (O’Neill et al., 2017). Thus, although it is possible that replay in deep MEC layers reflects replay of information from hippocampus, replay in the superficial layers of MEC may serve an as yet unknown function (Trimper et al., 2017).

In the context of memory consolidation, the direct output from CA1 to the prefrontal cortex (Jay and Witter, 1991) plays an important role (Takehara-Nishiuchi et al., 2006): (1) PFC engram cells can be re-activated optogenetically, and reactivation induces recall (Kitamura et al., 2017). (2) Performance of a spatial memory task induces increased hippocampal-PFC coupling in which optogenetic activation time-locked to hippocampal ripples in the post-task sleep epoch improves memory consolidation (Maingret et al., 2016). Importantly, such consolidation is not limited to areas monosynaptically connected to the hippocampus (auditory cortex; Rothschild et al., 2017).

Besides projecting to retrohippocampal areas and PFC, CA1 axons also extend to other brain areas (Groen and Wyss, 1990; Cenquizca and Swanson, 2006, 2007). But the bulk of the hippocampal outputs to cortical and diencephalic targets arises from the subiculum (Witter and Groenewegen, 1990; Kim and Spruston, 2012). The axons of individual subicular neurons extend to several target areas (Cembrowski et al., 2018), consistent with the idea that the subicular activity transforms the sparse spatial code in area CA1 (Thompson and Best, 1989) into a dense, distributed, redundant representation in the target areas (Kim et al., 2012). This organization makes it possible for any subset of output neurons to convey the same information to different downstream targets. While this has important implications for memory consolidation during slow wave sleep, very little is known about replay or slow wave sleep rhythms in the subiculum (Isomura et al., 2006). Nevertheless, the prime target of the subiculum, the retrosplenial cortex, can show learning-dependent, and memory-retrieval-predicting memory engrams, similar to those described above for PFC (Cowansage et al., 2014; Milczarek et al., 2018; see also Maviel et al., 2004).

### Up and Down States in the Hippocampus

As mentioned above, the generation of SWRs in CA3 (and downstream area CA1) is important for replay and memory consolidation. The circuit mechanisms that link SWRs to cortical Up and Down states are only dimly understood. Do cortical Up and Down states, particularly in the entorhinal cortex, affect activity in the hippocampus?

In contrast to both entorhinal cortex and somatosensory cortex, the hippocampus does not display clear epochs of Up and Down states; instead, hippocampal LFP activity during slow wave sleep has traditionally been characterized as “irregular” (for an early review of hippocampal activity, see O’Keefe and Nadel, 1978). Intracellular recordings in urethane-anesthetized mice (Hahn et al., 2007) and rats (Isomura et al., 2006) revealed unimodal, not bimodal, distributions of the membrane potential of hippocampal pyramidal neurons in CA1 and CA3 (Figure 1Ci). There was no clear depolarization related to Up states. The activity of granule cells in the dentate gyrus (DG), the target of entorhinal inputs via the perforant pathway, is skewed and only somewhat bimodal (Isomura et al., 2006; Hahn et al., 2007), likely reflecting a more direct and powerful cortical input to this structure. Finally, consistent with the extensive neocortical, paleocortical, and thalamic connectivity of the subiculum (Ding, 2013), the membrane potential of pyramidal cells in the subiculum, the principal output structure of the hippocampus, is clearly bimodal (Isomura et al., 2006).

Here for the sake of brevity, we focus on hippocampal area CA1. Although, as mentioned above, CA1 does not show the typical bimodal membrane potential distribution described for other cortical areas, neurons in CA1 do receive input from the MEC (Figure 1Civ), and MEC neurons display Up and Down states in their membrane potential. Bimodality in the neocortical membrane potential arises from some as yet undefined combination of recurrent connectivity, short term plasticity mechanisms, thalamic inputs, and intrinsic voltage gated conductances. Note that even if the membrane potential of single neurons is not bimodally distributed, inputs during each Up state could still increase membrane potential noise and trigger action potentials. Indeed, the firing of individual pyramidal neurons in CA1 is coupled to Up states recorded in S1 in both mice and rats (Sirota et al., 2003). Spikes in CA1 neurons occur just after the trough of S1 delta waves, just a bit after spikes in S1 units. This suggests that the synchronous cortical activity in an Up state can drive CA1. The firing of CA1 pyramidal neurons and the membrane potential of these neurons is also coupled to delta waves and Up-states in other cortical areas in the rat, including entorhinal cortex (Isomura et al., 2006; Figure 1Cii).

But the coupling of CA1 multi-unit activity reflecting the activity of CA1 pyramids, was more strongly modulated by the “persistent” Up states in MEC layer 3 than by Up states recorded in other neocortical areas (Hahn et al., 2012; Figure 1Bii). This coupling is consistent with the fact that axons from layer 3 pyramidal cells directly innervate CA1 pyramidal cells (Figure 1Civ). The depth of UDS modulation of the firing rates of CA1 pyramidal cells also appears to be subtype-specific: modulation strength differed between two types of CA1 pyramidal cells, identified by their sublayer-location within the pyramidal cell layer (Mizuseki et al., 2011). Finally, the LFP in CA1 stratum lacunosum moleculare (SLM) also reflects the influence of EC inputs, which impinge in this layer in CA1 and drive gamma oscillations locked to local slow oscillations and to the MEC Up state (Isomura et al., 2006; Wolansky et al., 2006; Sullivan et al., 2011).

So why is the cortical input to CA1 pyramidal cells not sufficient to induce large depolarizations in each Up state? The absence of a strong thalamic inputs could play a role. However, this is a feature the hippocampus shares with the entorhinal cortex (Figure 1Biv) and, as pointed out above, entorhinal pyramidal cells display bimodality in their membrane potential (Figure 1Bi) and can locally generate Up-Down states. The difference between CA1 and entorhinal or neocortical circuits, could be related to the stratified organization of inputs in CA1, where entorhinal axons are restricted to the SLM and contact the distal apical tufts of CA1 pyramidal cells. It could also be due to the paucity of recurrent excitatory connections between CA1 pyramidal cells (<1% of recorded pairs; Knowles and Schwartzkroin, 1981; Deuchars and Thomson, 1996; Figure 1Civ). This is in contrast to the neocortex, where one organizing feature of neocortical pyramidal neurons is that all pyramidal neurons have a local recurrent excitatory axon, providing excitation to pyramidal neurons and to the local inhibitory interneurons. Note, however, that recent work suggests extensive recurrent connections along the longitudinal axis of the hippocampus (Yang et al., 2014). Finally, the difference between CA1, entorhinal and neocortical circuits could also be related to intrinsic properties, for example persistent activity of CA1 pyramidal cells appears to be supported by intrinsic mechanisms (Knauer et al., 2013).

### Inhibition and Up and Down States in the Hippocampus

The axons from entorhinal L3 pyramidal cells contact CA1 pyramidal cells and interneurons, including those located at the border of stratum radiatum (SR) and SLM. These interneurons fire in phase with neocortical UP states (Hahn et al., 2006; Figure 1Ciii) and could be a source of feedforward inhibition to CA1 pyramidal cells (Vida et al., 1998). Interneurons at the SR-SLM border also receive inputs from pyramidal neurons in MEC layer 2 in mice (Kitamura et al., 2014) and express either nNOS, suggesting they include neurogliaform cells (Price et al., 2005), or calbindin, suggesting dendritic-layer-innervating or performant-path-associated CCK interneurons (Klausberger et al., 2005; Lasztóczi et al., 2011). CCK interneurons in turn can also innervate DG. Whether all classes of interneurons in CA1 fire in phase with Up and Down states has not been established (Isomura et al., 2006).

In general, global brain states modulate the level and type of activity of many interneuron types: (1) juxtacellular recordings show that SST-expressing O-LM cells decrease their firing rates during sleep compared to their activity during awake behaviors (Katona et al., 2014). The sleep-related activity of the also SST-expressing bistratified cells was more heterogeneous. (2) parvalbumin-expressing basket cells on the other hand increase their firing rate during slow wave sleep, whereas Ivy cells, the most common interneuron type in CA1 (Fuentealba et al., 2008), were not modulated by global brain state (Lapray et al., 2012); (3) Calcium imaging of activity shows that somatostatin and parvalbumin cell activity decreases during slow wave sleep compared to the awake state (Niethard et al., 2016). One limitation of the calcium imaging approach is that the different SST-expressing (e.g., bistratified versus O-LM) and PV-expressing (e.g., basket vs. axo-axonic) interneuron types cannot be distinguished when Ca-sensor expression is driven by just a single genetic marker (Klausberger and Somogyi, 2008). Taken together, these studies suggest that during slow wave sleep the amount of dendritic inhibition is reduced when compared to the awake state (Niethard et al., 2017). It is possible that this reduction in inhibition is an important component enabling memory-specific upregulation of synapse formation.

To gain a deeper understanding of how the activity of different interneuron types is modulated a more sophisticated genetic tagging approach (e.g., Fenno et al., 2014) or single-cell methods like juxtacellular or whole-cell recording are necessary (Tukker, 2019), preferably from naturally sleeping animals (Averkin et al., 2016).

### Neuromodulation of Up and Down States in the Hippocampus

As in other cortices, the hippocampal slow oscillations and ripple initiation are modulated by the action of a variety of neuromodulators including acetylcholine (Wolansky et al., 2006; Vandecasteele et al., 2014), dopamine (McNamara and Dupret, 2017), serotonin (Ponomarenko et al., 2003; Ul Haq et al., 2016b), noradrenalin (Ul Haq et al., 2016a; Swift et al., 2018), and others (see Atherton et al., 2015; Buzsáki, 2015).

Here we focus on the role of acetylcholine because it has been shown to have a strong effect on SWR initiation. Optogenetic stimulation of ChAT-expressing neurons in the medial septum reduces the incidence of SWRs, and suppresses the power of slow oscillations in CA1 (Vandecasteele et al., 2014). Systemically administered cholinergic agonists abolish hippocampal slow oscillations, whereas antagonists enhance them (Wolansky et al., 2006). This is consistent with a general role for ACh from the basal forebrain in shifting overall sleep-state in the neocortex (Han et al., 2014; Xu et al., 2015). The precise circuit mechanisms underlying direct effects of acetylcholine in the hippocampus are only dimly understood. The cell-type-specific synaptic and non-synaptic effects of acetylcholine on pyramidal cells and interneurons are not clear (Teles-Grilo Ruivo and Mellor, 2013; Dannenberg et al., 2017). It should also be noted that the systemic effects of cholinergic agonists and antagonists, as well as the effects of optogenetic or chemogenetic manipulations of cholinergic neurons in the medial septum, could be indirectly mediated via local network effects in the medial septum, which also sends GABAergic and glutamatergic projections to the hippocampal formation, with which it is reciprocally connected (for recent reviews, see Tsanov, 2015; Müller and Remy, 2018).

**Box 2. Novel Methods and Considerations Related to Replay and Memory**

Novel methods are likely to increase our understanding of the role of hippocampal outputs in memory consolidation during sleep (Chen and Wilson, 2017; Maboudi et al., 2018). Currently, the mechanisms that drive replay, and the role of the different inhibitory interneurons and neuromodulators in this process are only dimly understood. Beyond the relatively short SWR events associated with replay and memory consolidation, the possible role of hippocampal outputs during slow wave sleep also remains unclear. In this respect, it might be relevant to make a distinction between *deep* sleep stages, where homeostatic mechanisms are more important, and *light* sleep stages, where synaptic potentiation and thus memory consolidation is more likely to occur (Genzel et al., 2014). Up and Down states differ during the different stages of sleep, in part due to differences in neuromodulator levels (Genzel et al., 2014; Krishnan et al., 2016), although particularly in rodents these differences have not been thoroughly investigated.

The levels of most neuromodulators also differ markedly between sleep and awake states. This may help explain why hippocampal replay during wakefulness differs from that during sleep: awake replay can also occur in reverse order, and is likely to have additional roles besides memory consolidation (Foster and Wilson, 2006; O’Neill et al., 2006; Diba and Buzsáki, 2007; Jadhav et al., 2012; Roux et al., 2017). It is important to emphasize that even within awake periods there are large differences between the active and the resting state (McGinley et al., 2015; Poulet and Crochet, 2018; Kay and Frank, 2019). SWRs and replay have been observed during sleep, and during active and resting awake states, but are likely to involve different mechanisms and play different roles, at least in part related to acetylcholine and dopamine levels (Atherton et al., 2015).

Although it is increasingly clear that Up and Down states are also present in awake animals (e.g., Petersen et al., 2003; Ferezou et al., 2007; Vyazovskiy et al., 2011; Senzai et al., 2019), the precise differences of UDS generation and propagation between various awake and sleep states remain to be elucidated. During sleep, different subclasses of UDS were recently shown to be relevant for the consolidation of neuroprosthetic skill learning: optogenetically driving slow wave oscillations in rat primary motor area M1 improved consolidation, whereas delta waves supported forgetting of the task (Kim et al., 2019).

Whether this is also the case for episodic memory remains to be seen. There are important differences in the consolidation of procedural versus declarative memory (Headley and Paré, 2017), including the role of the hippocampus (Squire and Dede, 2015). It is also helpful to distinguish between different types of declarative memory: semantic and episodic memory are likely to involve dopaminergic and noradrenergic systems, respectively (Duszkiewicz et al., 2019).

## Summary and Outlook

Spontaneous activity in cortical circuits is generated internally during sleep and in quiet wakefulness, and propagates through the brain linking a variety of cortical and subcortical structures. While the links between neocortex, entorhinal cortex and hippocampus persist during the different brain states, the functional consequences of the activity change from state to state. In the awake state sensory information propagates to entorhinal cortex, and hippocampus, and during slow wave sleep and sharp wave ripples, the memories of the day are consolidated in neocortex with a replay of the day’s activity. To establish how this actually occurs, experiments imaging and manipulating the activity of axons that provide cortical and subcortical inputs to hippocampus during sensory information acquisition states and during replay will be necessary (Suh et al., 2011; Kaifosh et al., 2013; Kitamura et al., 2017; Yamamoto and Tonegawa, 2017; Qin et al., 2019), ideally in combination with novel methods of analysis and new approaches differentiating between different types of memory and awake/sleep states (Box 2). It would be useful to also image the dendritic activity of classes of neurons during these different states, to establish whether the active properties of dendrites, or the occurrence of NMDA/calcium spikes (Palmer et al., 2012, 2014; Larkum, 2013; Bittner et al., 2015, 2017; Takahashi et al., 2016; Schmidt-Hieber et al., 2017; Sheffield et al., 2017) have a role in replay, or memory consolidation and memory formation. It is still too early to tell whether changing the coupling between dendrites and soma has a role in learning or memory (Suzuki and Larkum, 2020), and whether the hypothalamic sleep and wake promoting neurons that are linked to other neuromodulatory systems and to neocortex have a role in the dialogue between hippocampus and neocortex (Zolnik et al., 2020).

In this review, we examined how the simple slow rhythm of the Up and Down states propagates from neocortex to hippocampus and back. A deeper understanding of the hippocampo-cortical dialogue and the relation between slow oscillations and memory consolidation is likely to be important for translational approaches targeting several brain disorders, including schizophrenia (Phillips et al., 2012), Alzheimer’s disease (Mander et al., 2015), and epilepsy (Gelinas et al., 2016). However, as is obvious from this review, the complete story of the interaction between these areas during sleep (and wakefulness) and the mechanisms that trigger memory consolidation is still to be told.

## Author Contributions

The initial concept of this review manuscript was conceived by RS and PB and discussed by all authors. JT, PB, and RS wrote the initial manuscript. JT prepared the figure. All authors edited and discussed.

## Conflict of Interest

The authors declare that the research was conducted in the absence of any commercial or financial relationships that could be construed as a potential conflict of interest.
